# Examining Lurasidone Efficacy in Patients with Schizophrenia Spectrum Illness and Concurrent Alcohol and Substance Use Disorder: A Prospective, Multicentric, Real-World Investigation

**DOI:** 10.3390/jcm13082206

**Published:** 2024-04-11

**Authors:** Clara Cavallotto, Stefania Chiappini, Alessio Mosca, Giacomo d’Andrea, Francesco Di Carlo, Tommaso Piro, Ottavia Susini, Giulia Stefanelli, Andrea Di Cesare, Valerio Ricci, Maria Pepe, Luigi Dattoli, Marco Di Nicola, Mauro Pettorruso, Giovanni Martinotti

**Affiliations:** 1 Department of Neurosciences, Imaging and Clinical Sciences, “G. D’Annunzio” University, 66100 Chieti, Italy; clara.cavallotto@laconchiglia.srl (C.C.); giacomo.dandrea1993@gmail.com (G.d.); francesco.dic@hotmail.it (F.D.C.); tommasopiro19@gmail.com (T.P.); stefgiulia3@gmail.com (G.S.); luigidattoli4337@gmail.com (L.D.); mauro.pettorruso@hotmail.it (M.P.);; 2 School of Medicine, UniCamillus International Medical School University, 00131 Rome, Italy; stefaniachiappini9@gmail.com; 3 Department of Mental Health, ASL 02 Lanciano-Vasto-Chieti, 66100 Chieti, Italy; andreadc25@gmail.com; 4 Department of Psychiatry, “San Luigi Gonzaga” Hospital, University of Turin, 10124 Turin, Italy; 5 University Policlinic Foundation “A. Gemelli” IRCSS-Catholic University of the Sacred Heart, Largo Agostino Gemelli, 8, 00136 Rome, Italymarco.dinicola@policlinicogemelli.it (M.D.N.)

**Keywords:** dual disorder, atypical antipsychotic, schizophrenia spectrum disorder, substance use disorder

## Abstract

**Background**: Dual disorders (DD) entail the coexistence of a substance use disorder (SUD) and another mental health condition, often within psychotic and affective disorders. This study aims to evaluate lurasidone, an innovative atypical antipsychotic, in individuals diagnosed with schizophrenia spectrum disorder and concurrent comorbidities of alcohol use disorder/substance use disorder (AUD/SUD). **Methods**: A cohort of 23 subjects diagnosed with schizophrenia spectrum disorder and comorbid AUD/SUD underwent psychometric assessments at baseline (T0) and one-month (T1) post-lurasidone initiation. **Results**: Lurasidone exhibited significant reductions in psychopathological burden, evidenced by decreased total PANSS scores (*Z* = 2.574, *p* = 0.011). Positive symptoms, substance craving (VAS Craving; *Z* = 3.202, *p* = 0.001), and aggressivity (MOAS scale; *Z* = 2.000, *p* = 0.050) were notably reduced. Clinical Global Impression (CGI) scores significantly improved (*Z* = 2.934, *p* = 0.003). Quality of life enhancements were observed in SF-36 subscales (energy, emotional well-being, and social functioning) (*p* < 0.05) and Q-LES-Q-SF scale (*Z* = −2.341, *p* = 0.021). A safety analysis indicated lurasidone’s good tolerability, with only 8.7% reporting discontinuation due to side effects. **Conclusions**: This study offers initial evidence supporting lurasidone’s efficacy and safety in dual diagnoses, highlighting positive effects on psychopathology, substance craving, and quality of life. These findings emphasize the need for tailored, comprehensive treatment strategies in managing the complexities of this patient population.

## 1. Introduction

### 1.1. The History of Dual Disorders

Major psychiatric disorders like schizophrenia, depressive disorders, and bipolar disorders are comprehensive conditions that shape an individual’s identity, hinder their ability to function, and diminish their overall well-being. Among the numerous coexisting conditions that coincide with these disorders, substance use disorder (SUD) and alcohol use disorder (AUD) stand out as the most challenging to effectively manage. This condition is referred to as a dual disorder (DD): that is to say, the comorbidity of at least one substance use disorder (SUD) and another mental illness, that in most cases includes psychotic and affective clinical categories [1]. The first identification of DD was in the 1980s, and became frequent in 1990; since then, different definitions have been offered, and an unambiguous definition is missing. The World Health Organization (WHO)’s definition of DD is “the co-occurrence in the same individual of a psychoactive substance uses disorder and another psychiatric disorder” [2,3]. Currently, the illicit market in Europe exhibits a diverse range of substances that are being imported in substantial quantities. These substances are purer and available at high potency compared to previous times; this is also due to the increasing supply of synthetic drugs produced in Europe [4].

An important factor of complexity is underscored by the bidirectional causality within dual disorders. Substance misuse may precipitate the onset of mental illness, while mental illness can induce substance misuse. Moreover, a dynamic and reciprocal exacerbation between the two disorders exists, where each may intensify the severity and progression of the other one [4]. 

Several reviews and meta-analyses consistently indicate a significantly higher prevalence of comorbidity in comparison to the general population [5]. For example, approximately one out of every five individuals with an eating disorder will develop SUD at some point in their life, with one out of every ten currently meeting the criteria for SUD [6]. Similarly, evidence suggests a disproportionate impact of opioid use disorder (OUD) among individuals with schizophrenia, who are less likely to receive opioid agonist therapy and tend to have a worse prognosis [7]. Additionally, the prevalence of comorbidity with cannabis use disorder (CUD) is notably higher among individuals with bipolar disorder [8]. As a result, in individuals with psychiatric disorders, having a dual diagnosis was correlated with substantial social or psychopathological difficulties, including violence, decreased mental health-related quality of life, encounters with law enforcement, homelessness, and incarceration [9]. Likewise, among individuals with SUD, having a dual diagnosis was associated with social or psychopathological disadvantages such as diminished mental health-related quality of life, exposure to childhood trauma, childhood sexual abuse, specific drug use diagnoses, suicide attempts, medical problems, multiple SUD diagnoses, childhood neglect, recurring adult traumas, and reduced social support [9].

### 1.2. Real-World Settings

Regarding various psychopathological aspects associated with substance use, the most prominent is psychosis, especially if the use is represented by high-potency cannabis [10] and new/novel psychoactive substances (NPS) [11,12]. Numerous substances have the potential to induce psychotic symptoms, a condition referred to as *substance-induced psychosis*, which does have a corresponding diagnosis in the DSM-5 [13]. The foundational diagnostic criteria for this condition stipulate that the psychotic symptoms should be transient, directly attributable to the effects of substance intake, essentially precluding the possibility of their persistence over an extended temporal span [11,12,13].

In real-world settings, the evolution of the clinical overview is often slow, which makes a clear distinction difficult, and it requires a significant amount of time. In this scenario, where there are no guidelines, the clinician must choose medication based on clinical observations. In this choice, it is crucial to consider certain key traits of people with SUD/AUD: they often are young subjects, frequently with a good functioning level [14], a tendency toward poor therapeutic adherence, altered behavior patterns characterized by increased impulsivity, emotional dyscontrol, and craving [15,16,17,18,19]. The desired pharmacological profile in this context should possess the capacity to address psychotic symptoms, while also exerting an impact on the emotional aspect by stabilizing mood and mitigating impulsive behaviors. Additionally, it should have a minimal metabolic impact and be manageable in day-to-day treatment.

As with this, real-world studies, such as observational studies, play a crucial role in understanding the effectiveness, safety, and tolerability of medications, and factors influencing treatment response in diverse patient populations outside of controlled clinical trial settings, including specific patient populations (e.g., elderly, pediatric, or those with comorbid medical conditions or SUD) to assess the efficacy and safety in these groups. In this regard, a very recent real-world study has shown the effectiveness and safety of esketamine in twenty-six subjects diagnosed with treatment-resistant depression in comorbidity with substance use disorder [20]. Moreover, there is an increasing number of real-world studies investigating the use of antipsychotics in patients with comorbid substance use. Early investigations suggested that atypical antipsychotics such as clozapine, risperidone, aripiprazole, and olanzapine were effective in managing psychotic symptoms and reducing substance use in individuals with dual diagnoses. However, subsequent research has yielded mixed outcomes, indicating that the effectiveness of treatment can be influenced by individual patient characteristics and the type of SUD [3,21,22,23,24,25,26,27,28].

This highlights the importance of pinpointing precise and efficacious treatments within the framework of a dual disorder.

### 1.3. Lurasidone: A Potentially Effective Drug in Dual Disorder?

Lurasidone is one of the most recent atypical antipsychotics and exhibits a more distinct receptor profile than first-generation antipsychotics. On one hand, this molecule has a high affinity for serotonin 5HT-2A and dopamine D2 receptors, deemed responsible for antipsychotic effects [20]. On the other hand, it has a strong affinity for 5HT-7 receptors which should yield favorable effects on mood, cognitive functions, sleep regulation, and negative symptoms [29,30].

Furthermore, it demonstrates a moderate affinity for serotonin 5HT-1A receptors, and for adrenergic α2 receptors which contribute to the positive effects on mood and cognitive functions and act positively on anxiety by reducing it [29,30].

Concerning the tolerability profile, this drug exhibits a low affinity for H1 and M1 receptors, which translates to a decreased risk of weight gain, metabolic changes, sedation, anticholinergic side effects like dry mouth and constipation [30], and a minimal risk of QTc prolongation [31]. Furthermore, it has been associated with significantly lower levels of increases in blood glucose, triglycerides, and prolactin compared to other atypical antipsychotics [32]. The only commonly reported side effects of lurasidone are akathisia and drowsiness, but it is important to note that these side effects are related to the dose-dependent effect of the medication [30,33].

Another intriguing aspect of lurasidone is its extended half-life, which falls within the range of 18 to 31 h. Consequently, it can be administered as a once-daily dose that facilitates the patient’s management of the therapy [30].

Currently, lurasidone is in use in the treatment of schizophrenia and is also employed in the management of bipolar depression, addressing both the mood disturbances and psychotic features associated with this condition [29,34,35].

In this regard, a recent meta-analysis suggests that lurasidone exhibits effectiveness over placebo, resulting in enhancements in both positive and negative symptoms, as well as overall psychopathology [36]. Furthermore, this investigation emphasized the favorable tolerability of lurasidone, with negligible impact observed on body weight, glucose levels, and lipid profiles [36]. Follow-up studies spanning from 6 to 22 months have corroborated the sustained efficacy of lurasidone in the treatment of schizophrenia while maintaining minimal effects on body weight and metabolic parameters [37,38,39,40]. Additionally, a recent 26-week open-label study, focusing on lurasidone administered at doses ranging from 40 to 80 mg per day, has expanded upon these promising findings to include patients with schizophrenia in various Asian regions, such as Japan, Taiwan, Korea, and Malaysia [41].

As a result, due to its pharmacological characteristics, lurasidone is considered one of the most well-tolerated antipsychotic medications in recent research, and it is recommended for use in patients with diverse comorbidities [42,43]. However, to the best of our knowledge, the literature on lurasidone treatment is limited for individuals with schizophrenia and a co-occurring SUD. A recent investigation [35] presents data on lurasidone in DD, regarding four patients experiencing their first cannabis-induced psychotic episode who were treated with lurasidone (74–128 mg/day), showing an improvement in the clinical picture of psychosis, with the remission of positive and negative symptoms and an improvement in overall functioning. Conversely, another study appears to be more comprehensive, focusing on the use of lurasidone in young individuals with complex psychopathological conditions [44]. Notably, the study documents the beneficial effects of lurasidone in a 14-year-old with a history of alcohol, cannabis, and LSD abuse, along with behavioral issues (self-injurious behaviors) and psychotic symptoms, such as auditory hallucinations [44].

### 1.4. Aim of the Study

The primary aim of our investigation is to evaluate the effectiveness of lurasidone in individuals diagnosed with schizophrenia who also have comorbid AUD/SUD. As a secondary objective, this study will explore the safety and tolerance of lurasidone in patients with schizophrenia spectrum disorder concurrently experiencing comorbidities associated with alcohol and/or substance use disorder.

## 2. Materials and Methods

### 2.1. Participants and Recruitment Centers

In this prospective, multicentric, real-world investigation, a cohort of twenty-three subjects diagnosed with schizophrenia spectrum disorder (DSM-5) and comorbid AUD/SUD (DSM-5) were sequentially enrolled from various mental health institutions in Italy. The coordination center was the Hospital Psychiatric Diagnostic and Treatment Service of the University Hospital S.S. Annunziata in Chieti. Other centers involved were the Inpatient Psychiatric Center of Villa Maria Pia in Rome, the Day Hospital of Psychiatry and Drug Dependence of the University General Hospital ‘A. Gemelli’ in Rome, and the Psychiatry Outpatient Clinics at the University Hospital ‘San Luigi Gonzaga’ in Turin.

The specialized unit at the University Hospital S.S. Annunziata in Chieti, known as the Hospital Psychiatric Diagnostic and Treatment Service, is designed for the evaluation and care of individuals experiencing an acute episode of psychiatric illness. Patients admitted to this unit receive a thorough array of interventions, encompassing precise diagnosis and the development of individualized treatment plans. The team comprises psychiatrists, psychologists, psychiatric nurses, and other professionals in the mental health field. The interventions may consist of pharmacological therapies, in-depth psychiatric assessments, and, notably, the ongoing monitoring of patients.

The Inpatient Psychiatric Center at Villa Maria Pia in Rome is a specialized facility for psychiatric treatment, specifically catering to post-acute patients. It provides a 30-day hospitalization period, which can be extended to a maximum of 60 days. Post-acute patients are those requiring elevated care interventions to stabilize their clinical condition after experiencing an acute episode of illness. This category includes individuals discharged from the Hospital Psychiatric Diagnostic and Treatment Service or those, with less severe cases than hospital admissions, still in need of inpatient care. The center emphasizes medication monitoring and the development of a medium- to long-term therapeutic program.

The Day Hospital of Psychiatry and Drug Dependence at the University General Hospital ‘A. Gemelli’ in Rome and the Psychiatry Outpatient Clinics at the University Hospital ‘San Luigi Gonzaga’ in Turin are involved in a variety of activities focused on the evaluation, diagnosis, treatment, and care of individuals with mental health disorders and substance use disorders who do not require continuous hospitalization monitoring. These facilities typically consist of a multidisciplinary team of professionals, including psychiatrists, psychologists, and nurses. Psychiatrists conduct comprehensive clinical assessments to diagnose various mental health conditions, involving patient interviews, review of medical histories, and the use of standardized assessment tools. Additionally, these professionals prescribe and manage medications to address psychiatric symptoms, assess their effectiveness, and monitor any potential side effects.

### 2.2. Study Design and Treatment Information

The investigation centered on a cohort of 23 individuals diagnosed with schizophrenia spectrum disorder (DSM-5) and concurrent AUD/SUD (DSM-5). Qualified psychiatrists thoroughly assessed the participants, examining their documented medical history and treatment records.

The patient eligibility criteria were as follows: individuals aged between 18 and 65 years and diagnosed with schizophrenia spectrum disorder and concurrent alcohol and/or substance use disorder. Exclusion criteria encompassed patients with ECG abnormalities (e.g., QTc > 450 ms), those with alcohol and/or substance use disorder in remission (more than 3 months without symptoms), individuals experiencing acute intoxication from alcohol and substances, or those with severe suicidal ideation. Additionally, exclusion criteria included pregnant or lactating individuals and those with a severe physical illness or evidence of mental illness severely impeding cognitive capacity.

Participants who had not previously received antipsychotic drugs or had been without such medication for at least 2 weeks were started on lurasidone. Conversely, those already on other antipsychotic medications underwent a cross-tapering procedure with lurasidone. Lurasidone was prescribed at doses ranging from 37 mg once daily to 148 mg once daily, adjusted based on individual clinical response.

### 2.3. Study Procedures and Psychometric Assessments

Anamnestic data were gathered at baseline (T0), while psychometric evaluations were conducted at T0 and one month (i.e., T1) following the initiation of treatment. Psychiatric symptoms were assessed using the Positive and Negative Syndrome Scale (PANSS) [45] and Clinical Global Impression-Improvement (CGI-I) [46] scores. The intensity of cravings for alcohol and/or substances was measured using a 10 cm visual analogue scale (VAS) [47]. Moreover, the study included an assessment of the quality of life (Quality of Life, Enjoyment and Satisfaction Questionnaire/Q-LES-Q-SF) [48] and global health condition (Short Form Health Survey 36/SF-36) [49]. Furthermore, the evaluation of alcohol and substance-related aggressiveness was conducted utilizing the Modified Overt Aggression Scale (MOAS) [50]. 

Finally, an experienced psychiatrist meticulously assessed adverse events associated with the administration of lurasidone and documented them in the patients’ medical records.

### 2.4. Statistical Analysis

Statistical analyses were performed using SPSS 20.0 software (SPSS Inc., Chicago, IL, USA) and JASP for Mac (JASP version: 0.16.4; JASP Team, 2022). All tests were two-tailed, with a statistical significance level set at *p* < 0.05. Continuous variables are expressed as means ± standard deviation (SD) or median [range], while categorical variables are reported as average numbers and percentages. We used the Shapiro–Wilk test to assess the normality distribution of continuous variables. Since the distribution of psychometric scales was found to be abnormal, the Wilcoxon signed-rank test was used to study variations across the different time points (i.e., T0 vs. T1). The matched rank biserial correlation (rrb) was used as a measure of the effect size.

### 2.5. Ethics

All participants, upon receiving comprehensive details about the drug’s features, the prescribed dosage regimen, and potential side effects, furnished written consent with complete awareness and comprehension. If the participant enrolled in the study was under legal guardianship, the informed consent was signed in the presence of the guardian. Conversely, if the participant was not under legal guardianship, the informed consent was signed in the presence of a family member. Furthermore, patients were informed of their ability to withdraw their consent at any point.

The research received approval from the local ethics committee (protocol n. 7/9 April 2015), local institutional review boards, and national regulatory authorities, complying with local regulations. The study adhered to the guidelines of Good Clinical Practice and the principles outlined in the Declaration of Helsinki (1964) and its subsequent revisions [51].

## 3. Results

### 3.1. Baseline Characteristics 

The final analysis cohort comprised 23 subjects (17 males and 6 females; mean age 33.77 ± 11.49). Detailed sociodemographic and clinical data are thoroughly presented in Table 1. The participant group predominantly consisted of young male individuals, most commonly diagnosed with substance-induced psychosis. AUD emerged as the most prevalent addiction disorder, succeeded by cannabis use disorder. Notably, a significant portion of the subjects were identified as polysubstance users (43.5%), underscoring the severity of the patient population examined in this study. 

Concurrent therapies are delineated in Table 1. Regarding pharmacological treatment, 14 subjects (60.9%) were antipsychotic drug-free; hence, they were initiated on lurasidone. Conversely, nine patients (39.1%) were already undergoing antipsychotic therapy, so they underwent a cross-tapering process with lurasidone. Among the most frequently administered medications prescribed apart from lurasidone were antiepileptics and benzodiazepines (14/23, 60.9%), followed by antidepressants (12/23, 52.2%).

### 3.2. Changes in Psychopathological Domains from Baseline to One-Month Follow-Up

**Figure 1:**
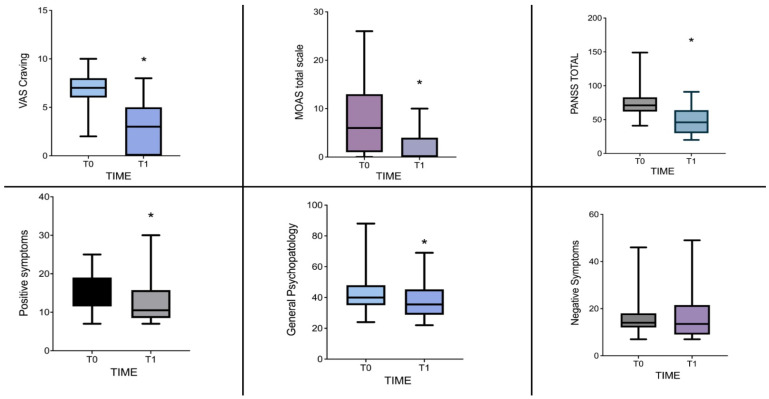
PANSS, MOAS, and VAS craving scores from baseline to one-month follow-up. * Indicates significant changes with a *p*-value < 0.05.

The Wilcoxon signed-rank test was used to evaluate the variations in psychometric scale scores from baseline to the one-month follow-up post-initiation of lurasidone treatment (i.e., T1) at a mean dosage of 85 mg/die (85.63 ± 46.94). As indicated in Table 2 and Figure 1, lurasidone effectively reduced the total PANSS score (*Z* = 2.574, *p* = 0.011) and the general psychopathology score, as measured with the PANSS subscale (*Z* = 2.605, *p* = 0.010). It also notably decreased positive symptoms in patients (*Z* = 2.132, *p* = 0.035), but did not significantly impact negative symptoms (*Z* = 0.471, *p* = 0.665). Importantly, from T0 to T1, there was a significant reduction in substance craving (VAS Craving, *Z* = 3.321, *p* = 0.001). Significant changes were also observed in aggressivity, as assessed by the MOAS scale (*Z* = 2.000, *p* = 0.050), and in the clinical global impression, as reflected by CGI scores (*Z* = 2.934, *p* = 0.003). Intriguingly, when comparing the effect sizes related to the reductions in different psychopathological dimensions one month after the introduction of lurasidone, craving was found to be the most significantly affected domain, exhibiting the highest effect sizes (rrb VAS for Craving = 0.971, indicating a significant effect size).

### 3.3. Changes in Global Health Condition and Quality of Life from Baseline to One-Month Follow-Up

**Figure 2:**
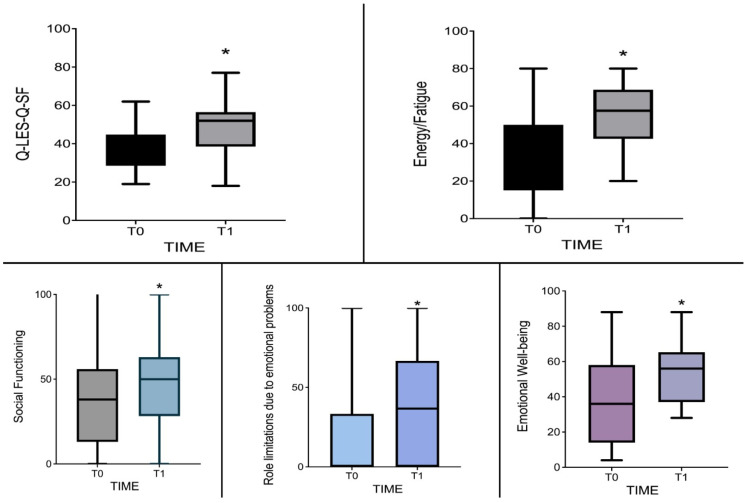
Q-LES-Q-SF and SF-36 scores from baseline to one-month follow-up. * Indicates significant changes with a *p*-value < 0.05.

A significant self-reported improvement in quality of life one month after initiating lurasidone treatment was observed, as evidenced by a notable increase in the 16-item Q-LES-Q-SF scale (*Z* = −2.341, *p* = 0.021) (Table 2; Figure 2). Additionally, the subscales of the SF-36 revealed a self-reported enhancement in energy (*Z* = −2.903, *p* = 0.004), emotional well-being (*Z* = −2.510, *p* = 0.013), and social functioning (*Z* = −2.432, *p* = 0.016) in the sample, accompanied by a decrease in limitations caused by emotional problems (*Z* = −2.521, *p* = 0.014) (Table 2; Figure 2).

### 3.4. Safety and Tolerability of Lurasidone

In the study, lurasidone was generally found to be safe and well tolerated. Notably, only 2 out of 23 patients (8.7%) reported significant side effects leading to their discontinuation of the treatment. These treatment-emergent adverse effects (TEAEs) resulting in discontinuation included sedation (1 out of 23) and extrapyramidal symptoms (1 out of 23).

The dropout rate was considerable, with 9 out of 23 subjects (39.13%) not completing the study. This included five subjects who were lost to follow-up, two subjects who discontinued treatment due to lack of efficacy and relapse into substance use, and the two subjects who withdrew due to TEAEs.

## 4. Discussion

As far as we know, this study provides insights into the efficacy of the atypical antipsychotic lurasidone in reducing psychopathological symptoms and substance craving and improving quality of life among individuals with psychotic symptoms and AUD/SUD.

Our study’s final analysis cohort comprised 23 subjects, predominantly composed of young males, consistently with epidemiologic data on dual diagnosis [52,53,54]. Patients were mostly affected with an AUD, followed by a cannabis use disorder, and a noteworthy portion of subjects were identified as polysubstance users (43.5%), underscoring the severity of the patient population studied, being data-coherent with the published literature [55,56].

Numerous individuals grappling with mental health challenges often turn to substance use, and individuals experiencing psychosis tend to exhibit a higher prevalence of problematic drinking and illicit drug use compared to the general population [57]. Despite ongoing studies regarding the establishment of a clear causal link between illicit drug use and the onset of psychosis [12,13], there is consensus on the detrimental impact of substance misuse on the trajectory of psychosis. This influence results in a more protracted and severe manifestation of the condition. The repercussions extend beyond the core symptoms, affecting various aspects of an individual’s life. Such consequences encompass a non-adherence to prescribed medication, a suboptimal engagement with treatment programs, a heightened suicide risk, an increased frequency of inpatient stays, an elevated potential for involvement in violent incidents, extended periods spent within the criminal justice system, and an overall compromised prognosis [16,58]. The limited studies available suggest potential benefits of antipsychotic treatment for this specific patient category [3].

The study assessed changes in psychopathological domains from baseline to a one-month follow-up after initiating lurasidone treatment, demonstrating its efficacy in treating psychotic symptoms and providing relief from hallucinations and delusions. Importantly, substance craving, measured by VAS craving, significantly decreased, and changes in aggressivity (MOAS scale) and CGI scores were also noted, suggesting mood-stabilizing effects overall improving quality of life, both with improvements in the subscales of the SF-36 (higher scores in energy, emotional well-being, social functioning, and reduced limitations caused by emotional problems) and an increase in the 16-item Q-LES-Q-SF scale. The beneficial effects of lurasidone on mood may have contributed to the normalization of ideational content while simultaneously alleviating dysphoric aspects present initially. Improvement in ideational and affective functions likely positively influenced the patient’s motivation, thereby mitigating psychopathological processes associated with substance abuse and craving. This, in turn, allowed for a more informed continuation of therapeutic progress and sustained abstinence from substances. Additionally, lurasidone demonstrates notable efficacy in addressing a cluster of symptoms including disorganized thinking, difficulty in comprehension and concentration, and poor memory. This effectiveness is evident in both clinical trial data and real-world observations [59,60,61,62]. This effect is primarily attributed to potent antagonism at serotonin 5-HT7 receptors and agonism at 5-HT1A receptors, both of which are recognized for their beneficial impact on cognitive function [63]. In turn, lurasidone’s specific pro-cognitive effects have the potential to enhance activity within the dorsolateral prefrontal cortex (DLPFC), a region commonly affected by substance use disorders. Recent trials have suggested that the targeted stimulation of this area could improve cognitive control over craving [64]. While acknowledging that this hypothesis may be considered speculative, the impact of lurasidone on craving could also be attributed to its enhancement of this mechanism.

Regarding safety and tolerability, lurasidone was generally found to be safe and well tolerated. Only 8.7% of patients reported significant side effects leading to treatment discontinuation, including sedation and extrapyramidal symptoms, which are instead effects reported as common with lurasidone [30].

However, the dropout rate was substantial, with 39.13% of participants not completing the study, including those who were lost to follow-up, those who discontinued due to lack of efficacy, those who relapsed into substance use, or those who withdrew due to treatment-emergent adverse events (TEAE). This observation aligns with previous research, encompassing reviews and clinical trials [16,65,66,67]. In this context, it is crucial to recognize the poor treatment adherence of dual-diagnosis patients as an inherent characteristic of this intriguing, yet complex, population.

### 4.1. Challenges and Considerations

The successful treatment of psychotic symptoms and AUD/SUD often involves an integrated approach that addresses both the psychotic symptoms and addiction concurrently and an overall risk of relapse, which can complicate the course of treatment and reduce related outcomes; this may involve the collaboration between mental health professionals and addiction specialists. Indeed, treatment plans need to be tailored to the individual’s specific needs, considering the severity of symptoms, the type of substances involved, and any other co-occurring medical conditions and monitoring for potential interactions. Mixing lurasidone and alcohol or other substances, especially central nervous system depressants, can intensify the effects of both, e.g., leading to increased drowsiness, sedation, and fatigue or impaired cognitive functioning and motor skills. The regular monitoring of substance use, which may increase the risk of relapse in individuals with psychosis, through regular psychiatric assessments, drug screenings, and collaboration with other healthcare providers, is essential. Moreover, individuals with a dual diagnosis may face challenges with medication adherence; in this regard, lurasidone is typically administered once daily; thus, ensuring its consistent use is crucial for its effectiveness. Providing education to patients about the potential risks and benefits of lurasidone, as well as the importance of medication adherence, can enhance treatment outcomes.

### 4.2. Limitations and Strengths of the Study

Our research, while valuable, encountered several noteworthy limitations that merit discussion and consideration. Primarily, the relatively limited number of participants included in our study emphasizes the importance of increasing the sample size. Expanding the participant pool would not only strengthen the statistical validity of our results but also improve the overall reliability and applicability of our findings. Secondly, the lack of comparison groups, particularly those comprising individuals treated with antipsychotics other than lurasidone or a placebo, presents a significant limitation to the comprehensiveness of our analysis. Including such groups in future studies would offer a more thorough insight into the comparative efficacy and safety of lurasidone in relation to alternative treatments. Another crucial aspect that needs addressing is the short duration of our study, which calls for further follow-ups to understand the long-term implications and sustained effectiveness of lurasidone. The use of an open-label study in our research, which may have influenced the outcomes and restricted the external validity of our conclusions, highlights the necessity of future investigations that utilize double-blind, randomized controlled trials, thereby yielding more robust evidence. Furthermore, in this intricate population, where polypharmacotherapy is frequently necessary, a potential confounding factor for our findings could be the use of other medications alongside lurasidone that might also be effective in treating SUD/AUD. Moreover, the multicentric nature of our study, conducted on individuals from different centers, could contribute to heterogeneous results. Finally, an additional limitation might be the lack of a standardized approach for evaluating the adverse events of lurasidone.

Nevertheless, our research possesses a notable strength: the non-randomized design of the study, which contributes to a closer alignment with clinical realities and represents a real-world scenario.

## 5. Conclusions

This study investigated the effectiveness of lurasidone in individuals with schizophrenia spectrum disorder and comorbid alcohol/substance use disorder.

The findings suggest that lurasidone treatment could result in a decrease in psychopathological burden, positive symptoms, and substance craving. However, the high rates of non-adherence emphasize the challenges in managing this population, highlighting the necessity for personalized treatment strategies and integrated mental health services. Although the study provides initial evidence supporting the efficacy of lurasidone in this complex patient sample, further research involving larger cohorts and longer follow-up periods is warranted.

## Figures and Tables

**Table 1:** Sociodemographic and clinical characteristics of the sample (*n* = 23).

Gender (male, %)	17 (69.56)
Age (y)	35.5 [18–54]
Substance abused	
Alcohol	13 (56.52)
Cocaine	8 (34.78)
Cannabis	12 (52.17)
Benzodiazepines	2 (8.7)
Methadone	1 (4.34)
Oxycodone	1 (4.34)
Amphetamines	1 (4.34)
Polysubstance users	10 (43.5)
Dual Diagnosis	
Schizoaffective Disorder	10 (43.48)
Substance-induced psychosis	13 (56.52)
Lurasidone dosages (mg)	74 mg [18.5 mg–148 mg]
Other therapies	
Antipsychotics	Olanzapine 5 mg/die, 1 (4.34) Promazine 30–120 mg/die, 5 (21.74) Quetiapine 100–200 mg/die, 2 (8.7) Tiapride 100 mg/die, 1 (4.34)
Antidepressants	Trazodone 50–150 mg/die, 9 (39.13) Sertraline 100 mg/die, 1 (4.34) Clomipramine 75 mg/die, 1 (4.34) Mirtazapine 30 mg/die, 1 (4.34)
Antiepileptics	Gabapentin 900–1600 mg/die, 7 (30.43) Lithium Carbonate 300–900 mg/die, 2 (8.7) Valproate 1500 mg/die, 2 (8.7) Pregabalin 300–450 mg/die, 2 (8.7) Lamotrigine 50 mg/die, 1 (4.34)
Benzodiazepines and Z-drugs	Alprazolam 7 mg/die, 1 (4.34) Delorazepam 1–4 mg/die, 4 (17.4) Diazepam 4–10 mg/die. 5 (21.74) Lorazepam 1 mg/die, 1 (4.34) Clonazepam 5 mg/die, 1 (4.34) Flurazepam 30 mg/die, 1 (4.34) Zolpidem 10 mg/die, 1 (4.34)
Others	Naltrexone 50 mg/die, 1 (4.34) Levomethadone 5 mg/die, 1 (4.34) Methadone 10 mg/die, 1 (4.34)

Data are presented as *n* (%), median [range], as appropriate.

**Table 2:** Changes in psychometric scales from baseline to one-month follow-up.

	Baseline (*n* = 23)	Follow-Up (*n* = 14)	*Z*	rrb	*p*
**PANSS**					
*Positive*	16 [7–25]	10.5 [7–30]	2.132	0.670	0.035
*Negative*	14 [7–46]	13.5 [7–49]	0.471		0.665
*General*	40 [24–88]	35.5 [22–69]	2.605	0.790	0.010
*Total*	71 [41–149]	60 [38–148]	2.574	0.781	0.011
**CGI**	6 [5–7]	3 [2–6]	2.934	0.328	0.003
**MOAS**	6 [0–26]	0 [0–10]	2.000	0.654	0.050
**VAS craving**	8 [2–10]	3.1 ± 2.5	3.202	0.971	0.001
**Q-LES-Q-SF**	38 [19–62]	2 [18–77]	−2.341	0.305	0.021
**SF-36**					
*Physical functioning*	90 [45–100]	95 [65–100]	−1.362		0.191
*Limitations due to physical health*	25 [0–100]	25 [0–100]	−0.770		0.482
*Limitations due to emotional problems*	0 [0–100]	36.67 [0–100]	−2.521	−1.000	0.014
*Energy/fatigue*	30 [0–80]	57.5 [20–80]	−2.903	−0.949	0.004
*Emotional well-being*	36 [4–88]	56 [28–88]	−2.510	−0.821	0.013
*Social functioning*	25 [0–100]	50 [0–100]	−2.432	−0.795	0.016
*Pain*	77.5 [0–100]	66.25 [20–100]	−0.118		0.953
*General health*	45 [0–75]	45 [5–85]	−1.117		0.255

Data are presented as median [range], as appropriate.

## Data Availability

The data presented in this study are available on request from the corresponding author due to privacy reasons.
